# Impact of congenital lower eyelid entropion correction surgery on pediatric astigmatism

**DOI:** 10.3389/fmed.2026.1822959

**Published:** 2026-04-14

**Authors:** Jing Wu, Yaxin Zhao, Nan Wei

**Affiliations:** Tianjin Key Laboratory of Retinal Functions and Diseases, Tianjin Branch of National Clinical Research Center for Ocular Disease, Eye Institute and School of Optometry, Tianjin Medical University Eye Hospital, Tianjin, China

**Keywords:** astigmatism, children, congenital lower eyelid entropion, corneal topography, retrospective study, surgical correction

## Abstract

**Objective:**

To evaluate the effect of congenital lower eyelid entropion surgery on refractive status and corneal astigmatism in children, and to explore surgical timing and visual rehabilitation.

**Methods:**

A total of 43 pediatric patients (43 eyes) aged 3–12 years who underwent entropion correction between June 2022 and June 2023 were included. Best-corrected visual acuity (BCVA), cycloplegic refraction and corneal topography were assessed preoperatively and at 1, 3, 6, and 12 months postoperatively. Statistical analyses included Spearman correlation, repeated-measures ANOVA, and nonlinear fitting.

**Results:**

Preoperative entropion severity correlated significantly with cylinder power (*r* = −0.583) and corneal astigmatism (*r* = 0.476). Cylinder power decreased progressively from −1.34 ± 0.84 D preoperatively to −1.04 ± 0.69 D at 6 months (*p* < 0.001). Steep corneal curvature (Ks) reduced from 44.41 ± 1.49 D to 43.85 ± 1.45 D at 6 months (*p* < 0.001), and further to 43.91 ± 1.28 D at 12 months (*p* < 0.001), while corneal astigmatism decreased from 1.82 ± 0.88 D to 1.42 ± 0.68 D at 6 months (*p* < 0.001) and remained stable at 1.42 ± 0.74 D at 12 months (*p* < 0.05). No significant change was observed in flat curvature (Kf). Refractive parameters stabilized by 3 months postoperatively, with the stabilization point identified at 3.06 months. Best corrected visual acuity (BCVA) improved from 0.85 ± 0.19 preoperatively to 0.95 ± 0.09 at 12 months (*p* < 0.001), and the proportion of amblyopic eyes decreased from 20.9% (9/43) preoperatively to 7.0% (3/43) at 1 year postoperatively.

**Conclusion:**

Surgical correction of congenital lower eyelid entropion significantly reduces astigmatism and improves visual acuity in children. Refractive status stabilizes by 3 months postoperatively, supporting early intervention to reduce amblyopia risk.

## 1 Introduction

Congenital lower eyelid entropion is a common eyelid developmental abnormality in Asian children, characterized by a horizontal crescent-shaped skin fold parallel to the lid margin. This redundant fold often extends beyond the lid margin and is frequently accompanied by epicanthus (1, 2). Redundant lower eyelid skin, orbicularis oculi muscle hypertrophy, and inadequate tarsal plate support can lead to lower eyelid entropion, causing eyelashes to turn inward toward the cornea and form trichiasis. These inward-turned eyelashes cause persistent corneal friction, resulting in symptoms such as photophobia, epiphora, and foreign body sensation. In severe cases, this may lead to corneal epithelial damage and erosion, affecting visual development (3, 4).

Recent studies have shown that pediatric patients with lower eyelid entropion exhibit significantly higher prevalence and degree of astigmatism than normal children (5, 6). Astigmatism is one of the most common refractive errors in children, and moderate-to-high astigmatism (≥2.00D) constitutes a significant risk factor for amblyopia (7). The mechanism by which lower eyelid entropion induces astigmatism may be associated with abnormal pressure on the eyeball from excess horizontal skin folds, blepharospasm triggered by eyelash irritation, and frequent eye rubbing by pediatric patients.

Given that entropion exhibits a self-correction tendency with age, traditional management prioritizes observation. Surgical intervention is typically considered only when significant corneal irritation symptoms or corneal lesions develop (2, 8). However, given the high incidence of lower eyelid entropion during the critical period of visual development (0–12 years) and its adverse effects on refractive development, whether refractive factors should be incorporated into surgical indications has become an urgent clinical issue to address.

Current research findings on whether corrective surgery for lower eyelid entropion can improve astigmatism remain inconsistent (9–11). These inconsistencies primarily stem from variations in study populations (e.g., age distribution at surgery, ranging from 2 to 12 years) (12), surgical techniques employed (e.g., modified Hotz procedure, rotating sutures, or skin-orbicularis muscle resection) (13–15), follow-up durations (varying from 3 to 24 months postoperatively) (16, 17), and the specific astigmatic indicators assessed (e.g., total cylinder power, keratometric values, or topographic astigmatism) (13). Consequently, the optimal surgical timing for achieving astigmatic improvement, the dynamic pattern of postoperative refractive changes, and the appropriate postoperative visual rehabilitation strategies remain to be clearly defined.

This study evaluated the impact of surgery on the refractive status of pediatric patients through retrospective analysis of clinical data from children who previously underwent corrective surgery for lower eyelid entropion, and explored the timing of surgery and postoperative visual rehabilitation strategies.

## 2 Research methodology

### 2.1 Study subjects

This study was designed as a retrospective analysis of clinical data. A total of 43 pediatric patients diagnosed with congenital lower eyelid entropion accompanied by trichiasis at the Strabismus and Pediatric Ophthalmology Clinic of Tianjin Medical University Eye Hospital between June 2022 and June 2023 were enrolled. All who underwent surgical treatment and completed 12 months of postoperative follow-up. Among the 43 patients, 41 had bilateral involvement and 2 had unilateral involvement. To avoid statistical bias arising from inter-eye correlation in patients with bilateral disease, one eye was randomly selected from each patient for analysis, resulting in a final sample of 43 eyes from 43 patients. As this was a retrospective study, no formal sample size calculation was performed; all eligible patients during the study period were included.

Inclusion criteria: (a) Diagnosis of congenital lower eyelid entropion with trichiasis, which may be accompanied by epicanthus; (b) Presenting punctate corneal lesions, erosion (positive corneal fluorescein staining), or accompanied by significant corneal irritation symptoms. Exclusion criteria: (a) Other congenital eyelid anomalies, corneal scars, congenital cataracts, strabismus, and other organic eye diseases; (b) History of ocular trauma or surgery; (c) Systemic organic diseases; (d) Uncooperative patients during examinations; (e) Patients without regular postoperative follow-up.

This study was conducted retrospectively, and the institutional review board approved the waiver of written informed consent from patients and their guardians. This study was approved by the Ethics Committee of Tianjin Medical University Eye Hospital (2024KY-11) and adhered to the principles of the Declaration of Helsinki.

### 2.2 Data collection

Clinical data were retrospectively collected from the electronic medical record system of Tianjin Medical University Eye Hospital. A standardized data collection form was used to extract demographic characteristics (age and sex), preoperative and postoperative ophthalmic examination findings (including entropion severity, corneal fluorescein staining results, refractive status, and optical biometry measurements), surgical techniques performed, and follow-up outcomes. All extracted data were independently reviewed and verified by two investigators to ensure accuracy.

### 2.3 Examination items and surgical procedures

Patients underwent follow-up examinations at preoperative and 1, 3, 6, and 12 months postoperative. Examination items included:

a) Uncorrected distance visual acuity and best-corrected visual acuity (BCVA) using standardized logarithmic visual acuity charts.
b) Slit-lamp microscopy to examine anterior segment structures and assess the severity of lower eyelid entropion according to the Khwarg grading scale (18).
c) Cycloplegic refraction: After achieving adequate cycloplegia with 0.5% compound tropicamide eye drops, retinoscopy was performed to record spherical power (DS), cylinder power (DC), and axis. The spherical equivalent (SE = DS + DC/2) was calculated.
d) Corneal topography examination: The Pentacam anterior segment analysis system was employed to measure steep corneal curvature (Ks), flat corneal curvature (Kf), and corneal astigmatism (AST). Cylinder power was converted into J0 and J45 components using Thibos vector analysis (19).

J0=−0.5×DC×cos(2×axis);

J45=−0.5×DC×sin(2×axis).

e) Diagnostic Criteria for Amblyopia: According to the Expert Consensus on the Prevention and Treatment of Amblyopia in Chinese Children published in 2021 (20), the diagnostic criteria for amblyopia are as follows: ① Best Corrected Visual Acuity (BCVA) is below the lower limit of the normal range for the corresponding age: the lower limit of normal visual acuity is 0.5 for children aged 3–5 years and 0.7 for children aged 6 years and above; ② The interocular difference in BCVA is ≥2 lines (on a logMAR chart); ③ Ocular organic pathologies are excluded. A diagnosis of amblyopia is made if any of the above criteria are met. In this study, the diagnosis of amblyopic eyes was based on BCVA measured after cycloplegic refraction, with exclusion of other organic ocular diseases such as strabismus, congenital cataract, and corneal scars.
f) Postoperative amblyopia treatment: None of the children received additional amblyopia training after surgery. At the 1-month postoperative follow-up, refractive correction was performed based on the results of cycloplegic refraction, and glasses with appropriate prescription were prescribed. For patients with a confirmed diagnosis of amblyopia prior to surgery, new glasses were prescribed after refractive stability was achieved at the 3-month postoperative follow-up, based on the results of the refraction examination. Patients were instructed to wear their glasses consistently and to attend regular follow-up visits (every 3–6 months). During this study, none of the children received active amblyopia treatment measures such as occlusion therapy, pharmacological suppression, or amblyopia training devices. The improvement in best-corrected visual acuity postoperatively reflects the combined effects of surgical relief from mechanical eyelid compression, restoration of corneal morphology, and correction of refractive errors through appropriately prescribed glasses following stabilization of refractive status.

All surgical procedures were performed by the same senior pediatric ophthalmologist. Surgical technique: Under general anesthesia, redundant lower eyelid skin was excised 5 mm below the eyelash margin. The orbicularis oculi muscle was anchored to the lower eyelid retractors with mattress sutures at the medial one-third junction of the eyelid margin. The incision was closed with continuous intradermal sutures. Standard postoperative dressing changes and suture removal were performed, followed by follow-up observation.

### 2.4 Statistical analysis

Statistical analysis was performed using SPSS software version 23.0. Measurement data are expressed as mean ± standard deviation (x̄ ± s). Spearman rank correlation analysis was employed to examine the correlation between entropion severity and refractive parameters. Repeated-measures ANOVA was used to compare refractive parameter differences across preoperative and postoperative time points. Paired *t*-tests were applied for preoperative versus postoperative comparisons. Nonlinear fitting was utilized to determine the stabilization time point for postoperative corneal astigmatism. Statistical significance was defined as *p* < 0.05.

## 3 Results

### 3.1 General demographic characteristics

A total of 43 children (43 eyes) with congenital epiblepharon who underwent surgical correction were included in this study. For patients with bilateral involvement (*n* = 41), one eye was randomly selected for analysis; for patients with unilateral involvement (*n* = 2), the affected eye was directly included. The mean age of the patients was 7.30 ± 2.71 years (range, 3–12 years). There were 23 male patients (53.5%) and 20 female patients (46.5%). The mean body mass index (BMI) was 18.72 ± 5.28 kg/m^2^, with 22 patients (51.2%) classified as normal weight, 9 (20.9%) as overweight, and 12 (27.9%) as obese.

According to the Khwarg classification, the severity distribution of epiblepharon was as follows: Grade I in 2 eyes (4.7%), Grade II in 17 eyes (39.5%), Grade III in 20 eyes (46.5%), and Grade IV in 4 eyes (9.3%). The preoperative refractive status showed a mean spherical equivalent (SE) of 0.15 ± 2.61 D, mean spherical diopter (DS) of 0.82 ± 2.69 D, and mean cylindrical diopter (DC) of −1.34 ± 0.84 D. Among the 43 eyes, 29 (67.4%) had astigmatism less than 2.00 D, and 14 (32.6%) had astigmatism of 2.00 D or greater. According to the Chinese Expert Consensus on Amblyopia Prevention and Treatment in Children (20). Among the 43 eyes, 9 eyes met the diagnostic criteria for amblyopia (Table 1).

**Table 1:** Summary of baseline characteristics in children undergoing lower eyelid entropion surgery.

Parameter	Total
Age (years)	7.30 ± 2.71
Gender (*n*, %)	
Male	23 (53.49)
Female	20 (46.51)
BMI (kg/m^2^)	18.72 ± 5.28
Normal (*n*, %)	22 (51.16)
Overweight (*n*, %)	9 (20.93)
Obesity (*n*, %)	12 (27.91)
Severity (eye, %)	
Grade I	2 (4.65)
Grade II	17 (39.53)
Grade III	20 (46.51)
Grade IV	4 (9.30)
Refractive status	
SE (D)	0.15 ± 2.61
DS (D)	0.82 ± 2.69
Eyes with CYL < 2D (%)	29 (67.44)
CYL ≥ 2D (eyes, %)	14 (32.56)
Amblyopia (eyes, %)	9 (20.93)

### 3.2 Correlation between the severity of lower eyelid entropion and refractive status

Spearman rank correlation analysis was performed to evaluate the association between epiblepharon severity and refractive parameters. The severity of epiblepharon showed no significant correlation with SE (*r* = 0.09, *p* = 0.408). A weak positive correlation was observed with DS (*r* = 0.27, *p* = 0.012). Notably, there was a strong negative correlation between severity and DC (*r* = −0.58, *p* < 0.001), and a moderate positive correlation with corneal astigmatism (AST) (*r* = 0.48, *p* < 0.001) (Table 2).

**Table 2:** Correlation analysis between severity of lower eyelid entropion and refractive status.

Parameter	Entropion severity	
	*r*-value	*p*-value
SE (D)	0.092	0.408
DS (D)	0.271	0.012^*^
DC (D)	−0.583	<0.001^**^
AST (D)	0.476	<0.001^**^

Spearman rank correlation analysis was employed. ^*^p ≤ 0.05, ^**^p ≤ 0.001. *r* denotes the correlation coefficient, with positive values indicating a positive correlation and negative values indicating a negative correlation.

### 3.3 Changes in refractive status during postoperative follow-up in pediatric patients with congenital lower eyelid entropion

**Figure 1:**
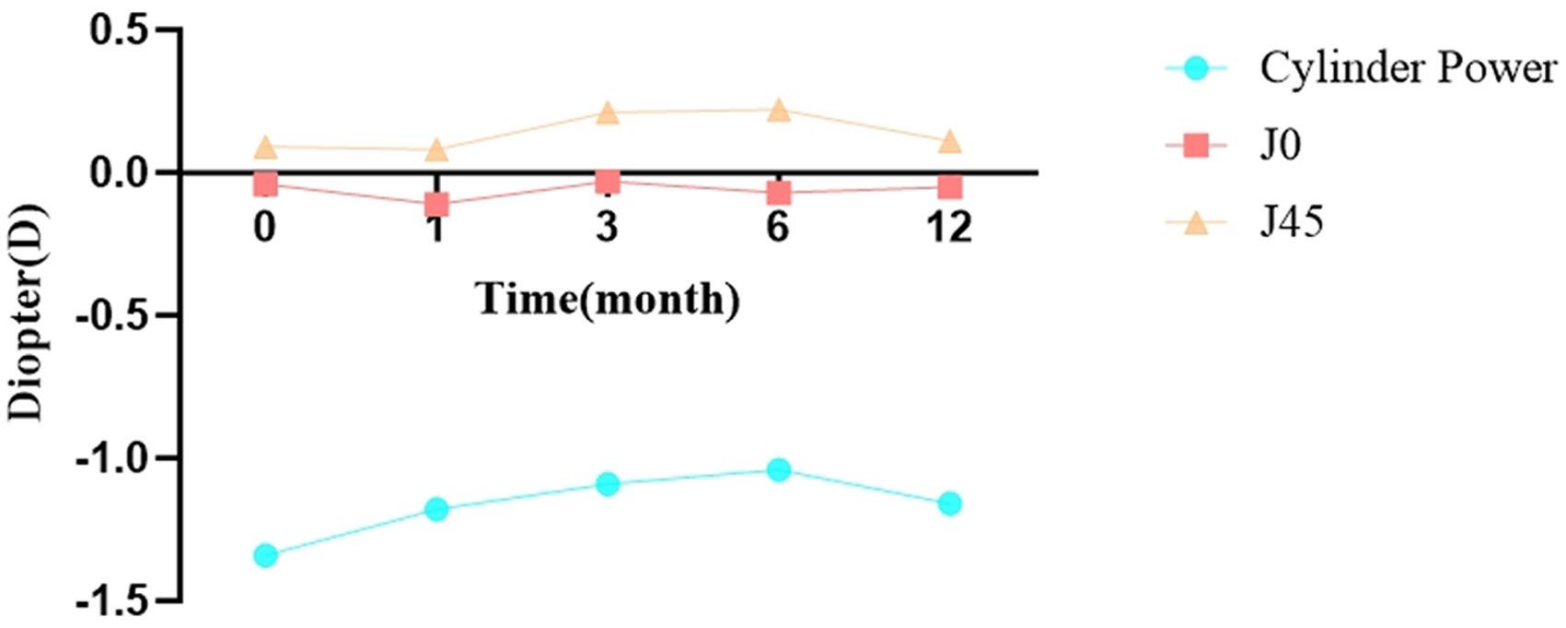
Changes in cylinder power over time following lower eyelid entropion correction surgery.

Patients were followed up at 1, 3, 6, and 12 months postoperatively. Cylindrical diopter (DC) decreased significantly after surgery: from −1.34 ± 0.84 D preoperatively to −1.18 ± 0.81 D at 1 month, −1.09 ± 0.73 D at 3 months, −1.04 ± 0.69 D at 6 months, and −1.16 ± 0.77 D at 12 months. The differences between preoperative values and each postoperative time point were statistically significant (*p* < 0.05 for 1 and 12 months; *p* < 0.001 for 3 and 6 months). Thibos vector analysis showed no significant changes in J0 or J45 over time (*p* > 0.05) (Table 3). Figure 1 illustrates the temporal changes in cylindrical diopter after surgery. The curve shows a rapid decrease during the first 3 months, followed by gradual stabilization. Nonlinear fitting analysis demonstrated that cylindrical diopter stabilized at 3.12 months postoperatively.

**Table 3:** Changes in cylinder power over time following lower eyelid entropion correction surgery.

Parameter	Preoperative	Postoperative 1 month	Postoperative 3 months	Postoperative 6 months	Postoperative 12 months
Cylinder power (D)	−1.34 ± 0.84	−1.18 ± 0.81	−1.09 ± 0.73	−1.04 ± 0.69	−1.16 ± 0.77
*p*-value	–	<0.001^**^	<0.001^**^	<0.001^**^	0.05^*^
J0	−0.04 ± 0.46	−0.11 ± 0.42	−0.03 ± 0.31	−0.07 ± 0.34	−0.05 ± 0.40
*p*-value	–	>0.05	>0.05	>0.05	>0.05
J45	0.09 ± 0.63	0.08 ± 0.55	0.21 ± 0.51	0.22 ± 0.44	0.11 ± 0.55
*p*-value	–	>0.05	>0.05	>0.05	>0.05

A paired *t*-test or Wilcoxon signed-rank test was used for comparison with preoperative values. ^*^p ≤ 0.05, ^**^p ≤ 0.001. CYL, Cylinder power; J0, J45, Thibos vector analysis parameters.

Corneal topography parameters were measured using Pentacam. Corneal steep keratometry (Ks) decreased significantly from 44.41 ± 1.49 D preoperatively to 43.98 ± 1.50 D at 1 month, 43.88 ± 1.48 D at 3 months, 43.85 ± 1.45 D at 6 months, and 43.91 ± 1.28 D at 12 months (all *p* < 0.001). Corneal flat keratometry (Kf) showed no significant changes except at 3 months (42.39 ± 1.66 D vs. 42.58 ± 1.69 D preoperatively, *p* < 0.001). Corneal astigmatism (AST) decreased significantly from 1.82 ± 0.88 D preoperatively to 1.53 ± 0.77 D at 1 month, 1.48 ± 0.73 D at 3 months, 1.42 ± 0.68 D at 6 months, and 1.42 ± 0.74 D at 12 months (*p* < 0.001 for 1, 3, 6 months; *p* = 0.002 for 12 months) (Table 4).

**Table 4:** Temporal changes in corneal astigmatism following lower eyelid entropion correction surgery.

Parameter	Preoperative	Postoperative 1 month	Postoperative 3 months	Postoperative 6 months	Postoperative 12 months
Ks (D)	44.42 ± 1.49	43.98 ± 1.50^**^	43.88 ± 1.48^**^	43.85 ± 1.45^**^	43.91 ± 1.28^**^
*p*-value	–	<0.001^**^	<0.001^**^	<0.001^**^	<0.001^**^
Kf (D)	42.58 ± 1.69	42.43 ± 1.64	42.39 ± 1.66^**^	42.43 ± 1.67	42.48 ± 1.59
*p*-value	–	>0.05	<0.001^**^	>0.05	>0.05
AST (D)	1.82 ± 0.88	1.53 ± 0.77^**^	1.48 ± 0.73^**^	1.42 ± 0.68^**^	1.42 ± 0.75^*^
*p*-value	–	<0.001^**^	<0.001^**^	<0.001^**^	0.02^*^

A paired *t*-test was used for comparison with preoperative values. ^*^p ≤ 0.05, ^**^p ≤ 0.001. Ks, Steep keratometry; Kf, Flat keratometry; AST, Corneal astigmatism.

**Figure 2:**
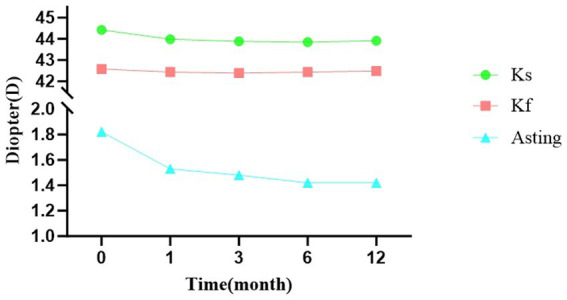
Temporal changes in corneal astigmatism following lower eyelid entropion correction surgery.

**Figure 3:**
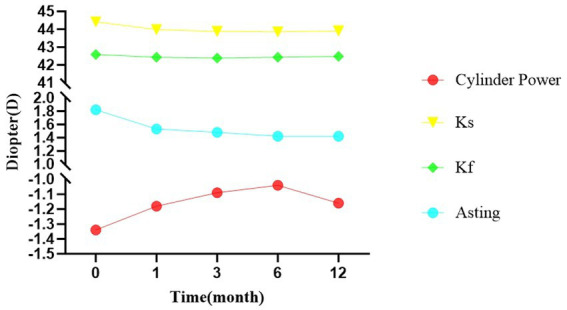
Postoperative stabilization point of corneal astigmatism in pediatric patients with lower eyelid entropion.

Figure 2 displays the changes in corneal topography parameters (Ks, Kf, and AST) over the 12-month follow-up period. The curve demonstrates that Ks and AST decreased rapidly during the first 3 months, while Kf remained relatively stable. Nonlinear fitting analysis revealed that corneal astigmatism stabilized at 3.12 months postoperatively, with Ks and Kf showing slight rebound after stabilization (Figure 3).

### 3.4 Changes in visual development during postoperative follow-up of pediatric patients with lower eyelid entropion

**Figure 4:**
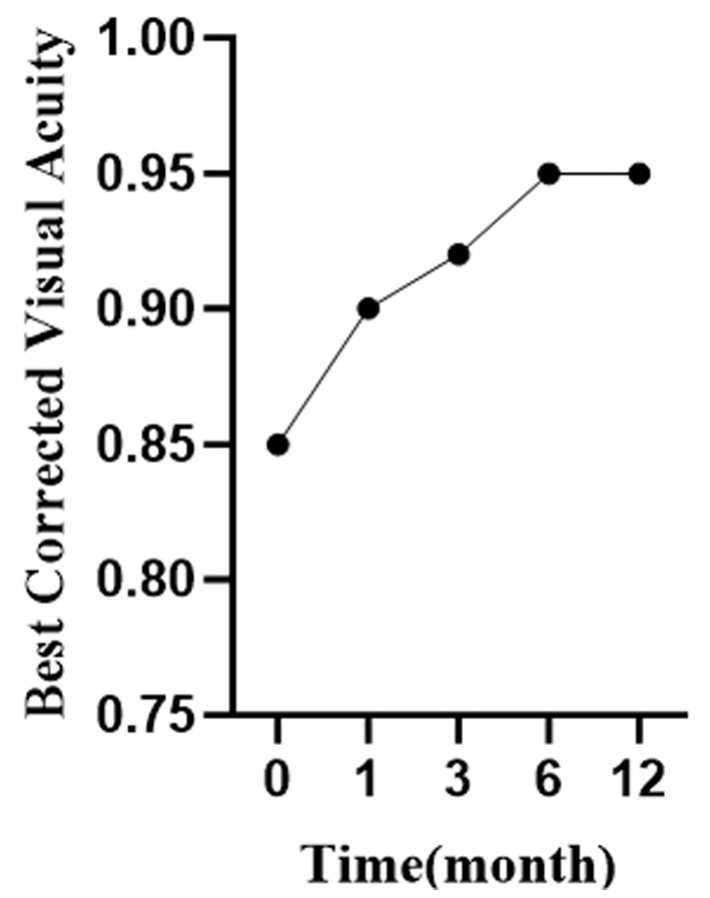
Curve of best corrected visual acuity changes over time following lower eyelid entropion surgery.

Best corrected visual acuity (BCVA) improved significantly after surgery. Preoperative BCVA was 0.85 ± 0.19, which increased to 0.90 ± 0.17 at 1 month (*p* = 0.002), 0.92 ± 0.13 at 3 months (*p* < 0.001), 0.95 ± 0.10 at 6 months (*p* < 0.001), and 0.95 ± 0.09 at 12 months (*p* < 0.001). Among the 9 amblyopic eyes preoperatively, only 3 remained amblyopic at the 12-month follow-up (Table 5). Figure 4 illustrates the improvement in best corrected visual acuity over time. The curve shows a progressive increase in BCVA during the first 6 months, reaching a plateau thereafter, indicating sustained visual function improvement following surgical correction.

**Table 5:** Curve of best corrected visual acuity changes over time following lower eyelid entropion surgery.

Parameter	Preoperative	Postoperative 1 month	Postoperative 3 months	Postoperative 6 months	Postoperative 12 months
BCVA	0.85 ± 0.19	0.90 ± 0.17	0.92 ± 0.13	0.95 ± 0.10	0.95 ± 0.09
*p*-value	–	0.02^*^	<0.001^**^	<0.001^**^	<0.001^**^

A paired *t*-test was used for comparison with preoperative values. ^*^p ≤ 0.05, ^**^p ≤ 0.001. BCVA, Best-corrected visual acuity (recorded as decimal acuity).

In this study, amblyopia was diagnosed according to the 2021 Chinese Expert Consensus on the Prevention and Treatment of Amblyopia in Children (20) best-corrected visual acuity (BCVA) of less than 0.5 in children aged 3–5 years, BCVA of less than 0.7 in children aged 6 years and older, or a difference of ≥2 lines in BCVA between the two eyes. Postoperatively, none of the pediatric patients received any amblyopia treatment other than refractive correction (e.g., occlusion therapy, pharmacological suppression therapy, or amblyopia training devices). The reduction in the number of amblyopic eyes and the improvement in best-corrected visual acuity reflect the combined effects of surgical relief from mechanical eyelid compression, restoration of corneal morphology, and correction of refractive errors through appropriately prescribed glasses postoperatively.

## 4 Discussion

Congenital lower eyelid entropion is a common eyelid developmental abnormality in Asian children, and its impact on refractive development is receiving increasing attention. This retrospective analysis of 43 pediatric patients undergoing corrective surgery for lower eyelid entropion demonstrated high preoperative prevalence and magnitude of astigmatism. Entropion severity showed significant correlations with cylinder power and corneal astigmatism (*p* < 0.001), suggesting that lower eyelid entropion may be an important contributing factor to pediatric astigmatism. The 12-month postoperative follow-up demonstrated that surgery not only effectively alleviated corneal irritation symptoms but also significantly reduced cylinder power and corneal astigmatism, improving best-corrected visual acuity. Moreover, refractive status tended to stabilize by postoperative 3 months.

This study found significant correlations between lower eyelid entropion severity and both cylinder power (*r* = −0.583, *p* < 0.001) and corneal astigmatism (*r* = 0.476, *p* < 0.001), indicating that more severe entropion is associated with a greater astigmatic error. These correlation coefficients were derived from a randomly selected single-eye analysis (*n* = 43) to avoid statistical bias from inter-eye correlation in patients with bilateral involvement. These results align with prior literature. Khwarg et al. (18) reports indicated that 35% of pediatric patients with lower eyelid entropion exhibited astigmatism ≥ 1.00 D. Shih et al. (21) observed that the degree of astigmatism increased with more severe corneal involvement or a greater number of trichiatric lashes. Our previous work (22) also demonstrated that pediatric entropion patients exhibit both higher mean astigmatism and increased prevalence of astigmatism. Similarly, Zhuo et al. (6) studied 3,170 children aged 3–6 years in Beijing found that those with lower eyelid entropion had a significantly higher risk of astigmatism (OR = 3.41) compared to their unaffected peers.

The mechanisms underlying astigmatism induction by lower eyelid entropion are not fully resolved, though several hypotheses exist. One hypothesis posits that redundant horizontal skin folds apply abnormal tension along the vertical corneal meridian, inducing with-the-rule astigmatism (23). Alternatively, astigmatic change may be attributed to eyelid squeezing or spasms caused by corneal irritation (24). Frequent blinking and eye rubbing due to trichiasis can also progressively alter corneal morphology (25). Furthermore, persistent corneal epithelial erosion may trigger apoptosis and cytokine release, promoting corneal remodeling and scar formation that ultimately result in astigmatism (26).

In the present study, postoperative analysis revealed a significant reduction in steep corneal curvature (Ks: from 44.41 ± 1.49 D preoperatively to 43.85 ± 1.45 D at 6 months, *p* < 0.001), while flat corneal curvature (Kf) remained largely unchanged (from 42.58 ± 1.69 D to 42.43 ± 1.67 D, *p* > 0.05). This finding supports the hypothesis that mechanical pressure from the eyelid along the vertical axis is a key contributor to astigmatic change. Surgical release of this pressure appears to flatten the vertical meridian and reduce astigmatism. Consistent with these observations, Lee et al. also reported a significant decrease in vertical corneal curvature within the 3-mm optical zone following entropion correction, with minimal change in the horizontal meridian—findings that closely align with those of the current study (27).

To rigorously evaluate the relative contributions of surgical intervention versus natural developmental changes to the observed astigmatic reduction, it is essential to consider the normative trajectory of astigmatism in healthy children. The present study included subjects aged 3–12 years, a critical period of visual development. Harvey et al. (28) conducted a longitudinal study of corneal astigmatism in 960 children aged 6 months to 8 years, demonstrating that from age 3–8 years, astigmatism decreased by only 0.11 D/year in the high astigmatism group (≥2 D) and 0.03 D/year in the low astigmatism group. More recently, Qin et al. (29) reported that in a large Chinese cohort of children with high baseline astigmatism (≥ 3.00 D), cylinder power decreased by only 0.048 D/year over a mean follow-up of 7.40 years. In the present study, based on randomly selected single-eye analysis (*n* = 43), the mean reduction in cylinder power was 0.30 D over 6 months (from −1.34 ± 0.84 D preoperatively to −1.04 ± 0.69 D at 6 months, *p* < 0.001), and the reduction in corneal astigmatism was 0.40 D over the same period (from 1.82 ± 0.88 D to 1.42 ± 0.68 D, *p* < 0.001). These reductions substantially exceed the normative annual change rates of 0.02–0.11 D/year reported in healthy pediatric populations (28, 29). Furthermore, the stabilization of refractive changes by postoperative 3 months in our study, with nonlinear fitting indicating stabilization at 3.06 months (95% of maximal change achieved), contrasts with the gradual changes seen in natural refractive development, suggesting a treatment effect beyond what would be expected from natural evolution alone. However, these comparisons are indirect, as they rely on external cohorts with different populations, methodologies, and baseline severity. The magnitude and rapidity of change exceed reported natural trends, suggesting a clinically meaningful effect of surgery.

Conclusions regarding whether corrective surgery improves astigmatism have been inconsistent across previous studies. In the present study, cylinder power decreased from −1.34 ± 0.84 D preoperatively to −1.04 ± 0.69 D at 6 months (*p* < 0.001) and −1.16 ± 0.77 D at 12 months (*p* < 0.05), while corneal astigmatism decreased from 1.82 ± 0.88 D to 1.42 ± 0.68 D at 6 months (*p* < 0.001) and 1.42 ± 0.74 D at 12 months (*p* < 0.05), supporting that surgery improves astigmatism. Notably, the most pronounced reduction occurred within the first 3 postoperative months, with parameters stabilizing thereafter. These findings align with previous studies. Park et al. (30) reported significant astigmatic reductions in children aged 5–7 years, those with amblyopia, and patients with unilateral entropion. Kim et al. (31) found significant astigmatism reduction at 3 months postoperatively, particularly in patients with high preoperative astigmatism and those aged 5–8 years. In their larger cohort, although overall astigmatism change was not significant, the high astigmatism subgroup (≥2.0 D) showed a marked decrease from 3.64 ± 1.77 D to 2.41 ± 1.03 D (*p* < 0.001)—closely aligning with our finding that 29.1% of patients had preoperative astigmatism ≥2.0 D and demonstrated significant postoperative improvement. Importantly, Kim et al. also reported that entropion surgery does not impair Meibomian gland function, with favorable safety profiles indicated by tear meniscus height, breakup time, and meibography, providing valuable support for clinical decision-making (31).

However, some studies did not find significant reduction in astigmatism after surgery. Ma et al. (9) observed that although postoperative astigmatism decreased in the surgical group, the difference was not statistically significant compared with conservative management (*p* > 0.05). Lee et al. (32) suggested that surgical indications should be based primarily on clinical manifestations rather than refractive considerations. Preechawai et al. (5) reported that mean astigmatism decreased from 1.28 D preoperatively to 1.19 D at 1 month postoperatively, but the difference was not significant, and no significant changes were observed during 1–2 years of follow-up. These inconsistent findings may be attributed to several factors: (a) Participant characteristics: variations in inclusion criteria, age distribution, and baseline astigmatism severity; (b) Surgical technique: differences in surgical approach, resection extent, and suture methods may affect the degree of corneal pressure relief; (c) Follow-up duration: astigmatism changes may manifest differently at various time points; (d) Measurement methods: differences in sensitivity and specificity between retinoscopy and corneal topography.

The study by Shindo et al. further enhances our understanding of surgical outcomes in pediatric entropion (11). Their findings confirmed that lower eyelid entropion surgery reduces corneal astigmatism, with an average decrease of 0.34 D at 6 months postoperatively in the incision group (*p* = 0.012). More importantly, the surgery significantly improved the Surface Regularity Index (SRI) and Irregular Astigmatism Index (IAI) (*p* < 0.01). Notably, 14.6 and 28.1% of patients were flagged as suspected keratoconus by corneal topography preoperatively; these proportions decreased significantly after surgery (*p* < 0.01). This suggests that entropion-induced corneal changes may mimic keratoconus, and surgical correction can reverse such topographic abnormalities, preventing unnecessary intervention.

You et al. provide novel evidence from the perspective of binocular visual function (33). In pediatric patients with concurrent lower eyelid entropion and strabismus, surgery led to significant improvements in stereopsis and fusion function. These findings indicate that entropion correction not only improves refractive status but also supports binocular visual recovery, offering additional rationale for surgical intervention in appropriately selected cases.

Synthesizing the latest research findings, the impact of lower eyelid entropion surgery on astigmatism may exhibit the following characteristics: (a) Surgical outcomes correlate with preoperative astigmatism severity, with pediatric patients experiencing high astigmatism demonstrating more significant improvements; (b) The surgery improved not only the degree of astigmatism but also corneal surface regularity and irregular astigmatism; (c) The surgery may ‘reverse’ some corneal topography abnormalities misdiagnosed as keratoconus; (d) The procedure demonstrated no adverse effects on Meibomian gland structure or function, indicating good safety; (e) The surgery may contribute to the recovery of binocular visual function.

This study found that changes in cylinder power and corneal astigmatism stabilized by 3 months postoperatively, with nonlinear fitting indicating stabilization at 3.06 months—a finding with important clinical implications. Astigmatism resulting from lower eyelid entropion is primarily attributed to prolonged mechanical compression on the cornea, analogous to the effect of orthokeratology lenses. As demonstrated by Yang Lina et al., corneal morphology requires at least 3 months to return to baseline after orthokeratology discontinuation (34). Given the chronic corneal compression in pediatric entropion, we recommend performing cycloplegic refraction and spectacle prescription no earlier than 3 months after surgery to avoid errors due to unstable refractive status.

This study found that amblyopic eyes decreased from 9 (20.9%) preoperatively to 3 (7.0%) at 1 year postoperatively, with significant improvement in best-corrected visual acuity (BCVA) (from 0.85 ± 0.19 to 0.95 ± 0.09, *p* < 0.001). All analyses used a randomly selected single-eye approach (*n* = 43) to avoid bias from inter-eye correlation in bilateral cases (41/43, 95.3%). Amblyopia was diagnosed according to the 2021 Chinese Expert Consensus on Amblyopia Prevention and Treatment in Children (20): for children aged 3–5 years, BCVA < 0.5 or interocular difference ≥ 2 lines; for children aged ≥ 6 years, BCVA < 0.7 or interocular difference ≥ 2 lines. All diagnoses were made after cycloplegic refraction, excluding organic ocular diseases. This limitation warrants prospective studies. Notably, the three children with persistent amblyopia at 1 year included two with preoperative severe amblyopia, underscoring that baseline severity is a key prognostic factor and suggesting the potential importance of earlier surgical intervention. No active amblyopia treatments (occlusion, atropine penalization, or amblyopia training) were administered postoperatively. Patients received only refractive correction with full-spectacle prescription at 1 month and re-prescription at 3 months after stabilization. The observed improvements likely reflect the combined effects of surgical eyelid pressure release, corneal recovery, astigmatism reduction, and subsequent refractive correction. However, because the study design did not include a control group receiving only refractive correction without surgery, the independent effect of surgery on amblyopia resolution cannot be definitively determined.

Visual improvement likely involves multiple mechanisms. Early BCVA gain (within 1 month) may reflect relief of corneal irritation and epithelial healing, while significant astigmatism reduction (cylinder: −1.34 ± 0.84 D to −1.04 ± 0.69 D, *p* < 0.001; corneal astigmatism: 1.82 ± 0.88 D to 1.42 ± 0.68 D, *p* < 0.001) may enhance retinal image quality and facilitate visual development. These improvements occurred without active amblyopia therapy, indicating that surgery with refractive correction alone can yield substantial visual benefits in children with congenital entropion and comorbid astigmatism.

Given the retrospective design, unrecorded amblyopia therapies cannot be entirely excluded, precluding differentiation between surgery alone versus combined intervention—a limitation warranting prospective studies.

The three children with persistent amblyopia at 1 year included two with preoperative severe amblyopia (BCVA < 0.2), highlighting baseline severity as a key prognostic factor and suggesting the potential benefit of earlier surgical intervention.

These findings align with previous reports. Yang et al. (17) reported that the proportion of eyes with BCVA worse than 5/9 decreased from 41.6 to 29% postoperatively. Kim et al. (31) reported improvement as early as 1 month, with greater gains in the entropion group versus controls. Cao et al. (11) found significant BCVA improvement at 6 months in children ≤6 years but not in older children, suggesting greater visual benefit from earlier correction. Collectively, these findings support early surgical intervention in children with congenital entropion and high astigmatism to prevent amblyopia onset and progression.

To address the potential statistical bias arising from inter-eye correlation in patients with bilateral involvement (41/43, 95.3%), we performed a randomly selected single-eye analysis (*n* = 43) as the primary analytical approach. Sensitivity analyses using generalized estimating equations (GEE) and separate analyses of left and right eyes confirmed the robustness of our findings. All three methods yielded highly consistent results: preoperative cylinder power ranged from −1.35 ± 0.85 D to −1.34 ± 0.84 D, correlation coefficients for severity with cylinder power ranged from −0.602 to −0.583, and postoperative stabilization times ranged from 3.06 to 3.15 months. These findings indicate that the main conclusions of this study are not influenced by the method of handling bilateral data.

Postoperative visual improvement may involve multiple mechanisms: enhanced corneal transparency due to resolution of mechanical friction from trichiasis and healing of epithelial erosions; improved tear film stability and distribution following normalization of eyelid morphology; reduced astigmatism leading to better retinal image quality; and optimized conditions for amblyopia therapy during the critical developmental period. These findings suggest that early surgical intervention in pediatric patients with congenital lower eyelid entropion and high astigmatism may not only reduce astigmatism but also support amblyopia prevention and treatment.

Traditional indications for entropion surgery include persistent corneal irritation unresponsive to conservative management, corneal epithelial damage, and corneal neovascularization or leucoma. However, as the relationship between lower eyelid entropion and refractive development becomes better understood, whether refractive considerations should be integrated into surgical indications has emerged as an important clinical question requiring further clarification.

This study, along with recent relevant research, provides evidence supporting the inclusion of refractive considerations in surgical decision-making. Pediatric patients with lower eyelid entropion have been shown to exhibit a higher prevalence and greater degree of astigmatism compared to healthy children (22), with entropion severity positively correlated with astigmatic magnitude. Corrective surgery effectively reduces cylinder power and corneal astigmatism, improves best-corrected visual acuity, and yields more pronounced benefits in children with high astigmatism. The procedure does not impair Meibomian gland function and demonstrates a favorable safety profile (10, 11). Additionally, surgery may reverse topographical abnormalities suggestive of keratoconus and promote recovery of binocular visual function. These findings collectively support expanding surgical indications to include refractive considerations in appropriate pediatric cases (11, 33).

Based on the evidence above, we recommend that surgical decisions for pediatric patients with congenital lower eyelid entropion should comprehensively consider clinical manifestations and refractive status: (a) For those presenting with significant corneal irritation symptoms or corneal lesions, surgical intervention should be considered regardless of the degree of astigmatism; (b) For patients without apparent corneal symptoms but exhibiting moderate-to-high astigmatism (≥ 2.00D), early surgical intervention may be considered to prevent the occurrence and progression of amblyopia; (c) For patients with lower degrees of astigmatism and no corneal symptoms, observation with regular follow-up of refractive status changes may be adopted; (d) For pediatric patients with amblyopia, lower eyelid entropion surgery should be considered prior to comprehensive amblyopia treatment to optimize refractive status.

This retrospective study has several limitations. Although we addressed the issue of inter-eye correlation by performing a randomly selected single-eye analysis (*n* = 43) with sensitivity analysis confirmation, the relatively small sample size limited statistical power for subgroup analyses. The 12-month follow-up may be insufficient to assess long-term refractive outcomes. The absence of a non-surgical control group makes it difficult to exclude the influence of natural ocular development on astigmatism reductions, though comparison with normative pediatric data suggests observed changes exceed expected variation. Reliance on medical records may introduce information bias. Surgical outcomes across age groups were not compared, leaving the influence of age on treatment efficacy to be clarified. Comprehensive ocular surface assessments (e.g., tear film, Meibomian glands) were not performed, leaving the impact on the ocular surface microenvironment unclear. Furthermore, as a retrospective study without a control group receiving only refractive correction, postoperative amblyopia management was not standardized or uniformly recorded; therefore, the relative contributions of surgery alone versus refractive correction alone to the reduction in amblyopic eyes (from 9 to 3 at 1 year) could not be distinguished. In addition, amblyopia diagnosis in this study was based on BCVA thresholds after cycloplegic refraction; in the absence of confirmatory testing for fixation behavior, binocularity, or compliance with glasses, some cases classified as amblyopia may have represented uncorrected refractive error. These limitations preclude definitive conclusions regarding the isolated effect of entropion surgery on amblyopia improvement and should be addressed in future prospective studies.

Future research should include multicenter, large-sample prospective cohort studies to better define the optimal timing and indications for surgical intervention. Advanced corneal imaging techniques, such as Scheimpflug imaging and optical coherence tomography, could be employed to investigate depth-dependent corneal changes following surgery. Extending follow-up through school age or adolescence would help clarify long-term effects on refractive development. Randomized controlled trials comparing surgical intervention with conservative management are needed to establish causality. Artificial intelligence-based prognostic models incorporating age, sex, BMI, baseline astigmatism, and entropion severity may support individualized surgical decision-making. Finally, health economic evaluations would be valuable to assess the cost-effectiveness of early surgical intervention.

Pediatric patients with congenital lower eyelid entropion exhibit a high prevalence of preoperative astigmatism, which correlates positively with the magnitude of astigmatism (*r* = −0.583 for cylinder power, *r* = 0.476 for corneal astigmatism, both *p* < 0.001). Corrective surgery alleviates mechanical corneal irritation and significantly reduces cylinder power (from −1.34 ± 0.84 D to −1.04 ± 0.69 D at 6 months, *p* < 0.001) and corneal astigmatism (from 1.82 ± 0.88 D to 1.42 ± 0.68 D at 6 months, *p* < 0.001), while also improving best-corrected visual acuity (from 0.85 ± 0.19 to 0.95 ± 0.09 at 12 months, *p* < 0.001). Postoperative refractive status stabilizes by 3 months (nonlinear fit stabilization point: 3.06 months), permitting reliable refraction and spectacle prescription at that time.

## Data Availability

The raw data supporting the conclusions of this article will be made available by the authors, without undue reservation.
